# The Effects of Dog Walking on Gait and Mobility in People with Parkinson Disease: A Pilot Study

**DOI:** 10.3390/ijerph17051549

**Published:** 2020-02-28

**Authors:** Suzanne O’Neal, Megan Eikenberry, Byron Russell

**Affiliations:** Physical Therapy Program, Midwestern University, Glendale, AZ 85308, USA; meiken@midwestern.edu (M.E.); brusse@midwestern.edu (B.R.)

**Keywords:** Parkinson disease, gait, balance, dual tasking

## Abstract

The objective of this study was to assess the effects of dog walking on gait and mobility in people with Parkinson disease (PD). This single-group, single-session, observational pilot study included nineteen participants with PD in Hoehn and Yahr stages II (*n* = 9) and III (*n* = 10). Primary measures were a gait analysis and the Timed Up and Go (TUG). Three trials of two conditions (walking with and without a dog) were completed. Walking with a dog resulted in slower gait velocity (mean difference = 0.11 m/s, *p* = 0.003, *d* = 0.77), shorter step length (left: mean difference = 7.11 cm, *p* = 0.000; right: mean difference = 3.05, *p* = 0.01), and stride length (left: mean difference = 7.52, *p* = 0.003; right: mean difference = 8.74, *p* = 0.001). The base of support was more narrowed (Z = −2.13, *p* = 0.03), with increased double limb stance time (left: Z = −2.89, *p* = 0.004; right: Z = −2.59, *p* = 0.01). Walking with a dog caused slower TUG times (mean difference = −1.67, *p* = 0.000) and increased number of steps (Z = −3.73, *p* = 0.000). No significant change shown in step time (left: mean difference = −0.001, *p* = 0.81; right: mean difference = 0.002, *p* = 0.77) or cadence (Z = −1.67, *p* = 0.10). In conclusion, there was an overall decline of gait parameters in people with PD when walking with a dog.

## 1. Introduction

Parkinson disease (PD) is a progressive, neurodegenerative disorder characterized by the cardinal signs of bradykinesia, tremors, rigidity, and postural instability. Gait disorders are typical consequences of PD, which include slowed speed, shortened stride length, and increased time in double limb support [1]. People with PD also exhibit increased gait asymmetry and loss of ability to maintain a steady gait rhythm. This can result in higher stride-to-stride variability [2]. These gait disturbances are the most significant motor complaint in advanced PD and can lead to an increased fall risk [3].

It has been shown that poor gait is associated with falls in the PD population [4]. It has also been established that gait abnormalities in people with PD can worsen under dual-task conditions [5]. Several studies have shown a significant decrement in gait characteristics, including a reduction in step length, stride length, and speed, when walking while performing a cognitive task [6,7,8,9]. Secondary motor tasks during walking have also been shown to have an effect on gait, with several studies showing degradation in several gait variables [6,7,8,10,11,12]. Secondary motor tasks can vary its effect on gait characteristics based on the complexity of the task. Bond and Morris [6] revealed that carrying an empty tray did not affect gait in people with PD, however, when four empty glasses were placed on the tray, this resulted in a significant decrease in gait speed and stride length. These gait changes seen under dual-task conditions have the potential to increase fall risk in people with PD.

Dogs are a common household pet in the United States (U.S.), with an estimated 36.5% of all U.S. households owning at least one dog [13]. Owning a dog has been shown to positively affect psychological health, including decreasing stress, anxiety, and depression, and increasing feelings of competence and self-esteem [14]. Dog walking has also been shown to promote physical activity, being associated with less disability and increased physical activity, among other health benefits [15,16,17]. Studies looking at dog owners and physical activity reported that dog owners had a weekly higher step-count than non-dog owners [18] and that dog owners were more likely to walk longer and more frequently than non-dog owners [19]. Dog owners were also shown to have faster walking speeds compared to non-dog owners [15]. Conversely, the presence of dogs in the household has also been shown to increase risk for falls [20] and, in fact, studies looking at reasons for falls due to owning a dog found that the act of walking a dog was the most common mechanism of falls leading to injuries [21,22]. To date, no literature exists which assesses the effects of dog walking on gait parameters in people with PD. The purpose of this study was to assess changes in gait parameters and the Timed Up and Go test (TUG) times between two conditions: (1) walking without a dog and (2) walking with a dog. The researchers hypothesized that the act of walking a dog would improve gait parameters in people with PD.

## 2. Materials and Methods

This study was a single-session, single-group, observational pilot study. This study was approved by the Midwestern University Institutional Review Board and written informed consent was obtained from all study participants. Nineteen people with a diagnosis of idiopathic PD participated in this study. It has been shown that a sample size of 12 per group for a pilot study is sufficient [23]; however, to accommodate for participants who did not meet criteria or did not attend the scheduled appointment, the researchers elected to schedule 20 participants. Inclusion criteria were diagnosis of idiopathic PD within stages II and III of the Hoehn and Yahr (H&Y) Rating Scale for PD, ability to walk at least 20 feet unassisted, and no aversions or allergic reactions to dogs. The H&Y scale is a descriptive rating scale of clinical function in PD, with stage I being of minimal functional disability and stage V being of severe functional disability [24]. Participants were excluded if they reported any other neurologic diagnosis, any current injury that would limit walking, and any uncontrolled cardiovascular issue. Participants were recruited by convenience sampling methods through local PD support groups and exercise classes. All inclusion/exclusion criteria except for the H&Y ratings, were screened either by phone or through email correspondence. Those who met the criteria were scheduled to attend a single research session.

**Figure 1:**
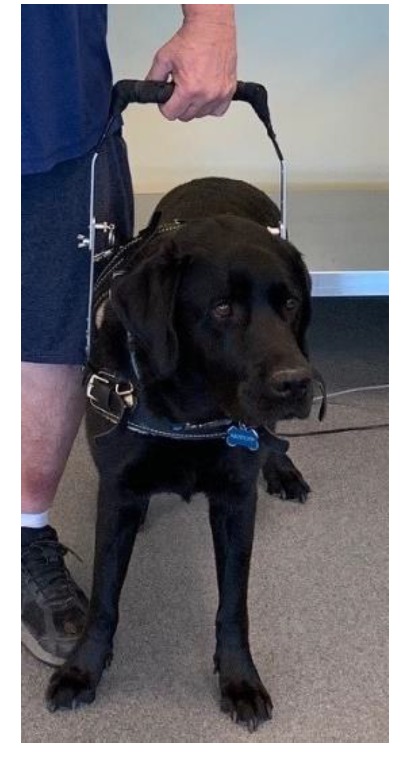
Trained service dog and rigid-handled harness used.

One dog was used for all participants. The dog was obtained by word of mouth. Inclusion criteria for the dog included documentation of all necessary vaccinations and history of obedience school or service-training. A labrador retriever that had completed service-training as a mobility and diabetic alert dog was selected for this study. A mobility harness with an attached, adjustable rigid handle was worn by the dog for all trials (Figure 1).

Each participant was scheduled for a one-hour time block on one of two data collection days. The one-hour time block start times were scheduled every 20 min. This allowed one participant to start on pre-testing while the other finished the completion of the research tests. The principal investigator (PI) first met with each participant to explain the details of the study, to obtain written informed consent, to obtain basic diagnosis-specific medical history, and to administer the Movement Disorders Society-sponsored revision of the Unified Parkinson’s Disease Rating Scale, Part III (motor examination subsection) (MDS-UPRDS-III). This occurred in a private room separate from the main research space. All participants were informally assessed for cognitive dysfunction during history taking and administration of the MDS-UPDRS-III. All participants were able to follow multi-step commands, were fully aware of the study protocol, and were able to sign their own informed consents. The PI was trained in the use of the MDS-UPDRS-III, having successfully completed the International Parkinson and Movement Disorder Society’s MDS-UPDRS Training Program and Certificate Exercise. All participants were able to follow multi-step commands; therefore, all completed the necessary components of the MDS-UPDRS-III. The information gained from the results of the MDS-UPDRS assisted the PI in determining the H&Y rating for each participant. All participants who attended the research session (*n* = 19) met the H&Y criteria, and therefore continued with the study. Once deemed eligible, the participant was then escorted to the main research space to continue with data collection. Total time to complete all tests was approximately 40 min.

The study took place in a canine therapy laboratory located in the Animal Companion Clinic at Midwestern University. The participants completed the TUG and a walking analysis using an instrumented walkway (GAITRite, CIR Systems, Franklin, NJ, USA). The order of the tests remained the same with each participant, with the TUG being first, and the gait analysis being second.

The TUG is a physical performance mobility test that is used in the PD population. It has been shown to have excellent inter-rater reliability (ICC = 0.87–0.99) [25], excellent test–retest reliability (r = 0.90–0.97) [26], and to be a predictor of fall risk [27]. Each participant was asked to complete the TUG under two conditions: walking without a dog (W-ND) and walking with a dog (W-D). The order of the two conditions was determined by a coin flip. During the W-ND condition, the participant was asked to start from a seated position in a standard-height chair. When the investigator said “go”, the participant stood up, walked around a cone placed three meters away, walked back to the chair, and sat down. The timer was stopped once the participant’s buttocks came in contact with the chair. Three trials were completed and the time and number of steps to complete each trial was recorded on a data sheet. During the W-D condition, each participant was asked to hold onto a rigid handle connected to a mobility harness worn by the dog. The participant was asked to select which hand to use to hold onto the harness handle. Following selection, the researcher then demonstrated the correct walking path around the cone that would allow the dog to remain on the outside of the walking path. For example, if the participant selected the left hand, the researcher would demonstrate walking around the cone, keeping the cone on the right side. This was done to ensure that the dog remained on the outside of the walking path to reduce the risk of the dog interfering with the cone. The participant was allowed to hold onto the handle prior to starting the test, but then was asked to let go of the handle prior to sitting back down. This was done to ensure that the dog would not come between the participant and the chair when sitting back down. Three trials were completed and the time to complete and number of steps to complete was recorded.

The GAITRite^®^ system is a portable walkway used for gait analysis to obtain objective data on spatiotemporal gait parameters [27]. The GAITRite^®^ system has good-to-excellent test-retest reliability (ICC = 0.79 to 0.98) [28], excellent concurrent validity with paper-and-pencil gait analysis methods on spatial measures (right step length ICC = 0.97; left step length, ICC = 0.99), and excellent concurrent validity with video-based methods on temporal measures in healthy populations (right step time ICC = 0.97; left step time, ICC = 0.95) [29]. A research assistant trained in the use of the walkway and software ran the program for each participant. The participants were asked to walk along the instrumented walkway during two conditions: W-ND and W-D. The order of the two conditions was determined by a coin flip. During the W-ND condition, participants were asked to walk along the walkway at their preferred, comfortable pace for three trials. Each trial was recorded and gait parameters were obtained through the walkway program. For the W-D condition, each participant was asked to hold the rigid dog harness with the left hand, due to the walkway having raised areas on the right side. This allowed the dog to walk uninterrupted. The participant was again asked to walk along the instrumented walkway at their preferred, comfortable pace, with the dog, for three trials. Each trial was recorded and gait parameters were again obtained from the walkway program. Results from trials of each condition were averaged. Gait variables included in this study were velocity, step length, stride length, base of support, cadence, % of gait cycle in double limb support, and step time.

Means of three trials for each condition for both the TUG and walking trials were analyzed and compared using statistical software (IBM SPSS Statistics for Windows, Version 25.0, IBM Corp., Armonk, New York). A Shapiro–Wilk analysis was run to determine normality of data. Normally distributed data were analyzed using a paired samples *t*-test. Data not normally distributed were analyzed using a Wilcoxon-signed ranks non-parametric test. To report effect sizes, Cohen’s d (*d*) was calculated for results of the paired samples *t*-test and a matched-pairs rank-biserial correlation (*r*) was calculated for results of the Wilcoxon-signed ranks test. To interpret Cohen’s *d*, the following effect size thresholds were used: small = 0.20, medium = 0.50, large = 0.80, very large = 1.30. To interpret *r*, the following thresholds were used: small = 0.10, medium = 0.30, large = 0.50, very large = 0.70.

## 3. Results

Twenty people were scheduled to take part in the study. Nineteen people with PD participated in this study, with one participant not attending their scheduled research time (Table 1). The mean age was 70.74 (7.19) years and the mean time since diagnosis was 5.07 (3.87) years. Participants were either in stage II (*n* = 9) or III (*n* = 10) of the Hoehn and Yahr disease rating scale. A Shapiro–Wilk analysis revealed several variables that were not normally distributed. These variables (BOS, left and right double limb support, number of steps during the TUG) were analyzed with a non-parametric Wilcoxon-signed ranks test (Table 2). Variables that were normally distributed (velocity, cadence, left and right step length, left and right stride length, left and right step time, and TUG) were analyzed using a paired samples *t*-test (Table 3). During gait on the instrumented walkway, walking with a dog resulted in significantly slower gait velocity (mean difference = 0.11 m/s, *p* = 0.003), nearing a large effect size (*d* = 0.77), significantly shorter step length (left: mean difference = 7.11 cm, *p* = 0.000, *d* = 1.05; right: mean difference = 3.05, *p* = 0.01, *d* = 0.65), and stride length (left: mean difference = 7.52, *p* = 0.003, *d* = 0.79; right: mean difference = 8.74, *p* = 0.001, *d* = 0.87). Walking with a dog also resulted in significantly slower TUG times (mean difference = −1.67, *p* = 0.000) with a very large effect size (*d* = 2.03). A non-parametric Wilcoxon signed-ranks test showed a significant increase in the number of steps during the TUG with dog (Z = −3.73, *p* = 0.000) with a large effect size (*r* = 0.61). The base of support was also significantly more narrowed (Z = −2.13, *p* = 0.03) with a medium effect size (*r* = 0.35). The time in double limb support also increased when walking with a dog (left: Z = −2.89, *p* = 0.004, *r* = 0.47; right: Z = −2.59, *p* = 0.01, *r* = 0.42). No significant change was shown in left or right step time (left: mean difference = −0.001, *p* = 0.81; right: mean difference = 0.002, *p* = 0.77) or cadence (Z = −1.67, *p* = 0.10).

## 4. Discussion

A large percentage of people living in the U.S. are dog owners, and owning a dog can result in a variety of emotional and physical benefits [14,15,16,17]. Owning a dog can also pose a fall hazard, especially in the elderly population, as the act of walking a dog can increase the risk of falls and injuries [21,22]. The ability to dual-task is also negatively affected as the disease progresses. The basal ganglia play a role in regulating movement, so that tasks such as walking becomes automatic [11]. When movements are automatic, this allows a person’s attention to be directed to other tasks, therefore maintaining the ability to dual-task [11]. When the basal ganglia are damaged, for example, in PD, movement patterns such as walking require increased attention, to bypass the damaged basal ganglia and utilize cortical regions [11]. This affects a person’s ability to attend to two tasks simultaneously. To date, no studies have investigated the effects of dog walking on gait in people with PD. People with PD commonly present with deterioration in motor function and worsening gait pattern, with slowed gait velocity, decreased stride length, and increased time in double limb support [1,30]. People living with PD continue to experience a progressive decline in these motor symptoms and this deterioration, among other factors, has been identified as a fall risk factor in the PD population [4]. In our present study, walking a dog further worsened these traits, with slower gait speed, decreased step length, decreased stride length, decreased base of support, and increased time in double limb support. In a normal gait cycle, double limb support comprises approximately 20% of the gait cycle [31] and people with PD typically present with increased time in double limb support compared to healthy individuals [1]. Our sample was consistent with this, presenting with an average of 29.2% in double limb support; however, walking with a dog continued to significantly worsen this to an average of 31.5%. The slower pace and shorter step length was evident with both the walking analysis and TUG results. Of the gait variables, the left step length demonstrated the greatest decline (10.69%), which may be attributed to the fact that the dog was positioned on the participants’ left sides, therefore causing more interference to the left step progression. The TUG demonstrated a 19.90% worsening of time. When walking without a dog, the mean time was 8.44 s, falling within the normal range of a healthy population in this age group (8–9 s) [32]. However, when walking with a dog, the mean increased beyond normal range (10.12 s), approaching the identified fall risk cut-off time of 11.5 s for people with PD [33].

To these researchers’ knowledge, no literature exists specifically identifying the motor and cognitive abilities needed to walk a dog, however, in theory, this task can be comprised of both a secondary motor and cognitive task. There is motor involvement of holding onto the dog leash, reacting to the dog’s movements, and producing movements to allow the dog to go on an intended path. There may be a cognitive involvement in that a person needs to be aware of the dog within the environment, to be aware of surroundings and potential distractions, and to plan the path of the dog. Prior to this study, the researchers hypothesized that the dog would improve gait parameters, including increased gait velocity and step length, however, the opposite was true. The participants in this study, when walking a dog, demonstrated declines in gait velocity, step length, and cadence with an increase in stance time. These results are consistent with previous studies assessing the effects of dual-tasking on gait variables [8,34]. Based on the design and results of this study, the authors are unable to confirm that the act of walking a dog caused deterioration in gait variables based on the dual-tasking nature of the task. The study’s results do, however, demonstrate that the added condition of walking a dog resulted in a significant decline in gait variables. This may bring to light the potential fall risk factor walking a dog may be to people living with PD, and that the act of walking a dog may not be as passive as it seems. Further studies are needed to specifically assess the act of walking a dog and the cognitive load it requires.

There are several limitations in this pilot study. First, this study lacked a control group, however this was intent as the aim of this pilot study was to assess effects of dog walking on gait changes specific to PD and not to compare changes to a normal population. It is well known that people with PD experience deterioration of gait, therefore the researchers were interested in whether there is further deterioration when walking a dog, which could increase fall risk and decrease overall safety. Secondly, several of the participants were able to ambulate faster than the dog, therefore leading the dog, instead of the dog leading the person. There was no way to ensure that the pace of the dog remained consistent with each participant, although recruitment of a service-trained dog minimized the variability in the dog’s pace. Third, we opted for a rigid handle to allow more control of the dog, especially for those who had significant upper extremity tremors, however, this type of lead may not be generalizable. Another limitation is that no formal cognitive testing was used in this study to correlate cognitive dysfunction to gait changes when walking a dog. Instead, in this pilot study, we opted to informally screen cognition and look at changes between the same group on two different conditions. This study was a single-session design over a relatively short walking path in a controlled environment. This controlled environment of a smooth, flat surface without distractions is not generalizable to community-level ambulation. However, this lack of distractions allowed the researchers to introduce the dog variable without other factors affecting the participants’ gait. Future studies that explore gait variability when walking longer distances over community terrain are needed. Additionally, the dog used in this study was a service-trained dog, and therefore walked along with good adherence to the path. This study’s results may have resulted in more dramatic changes in gait parameters if a non-trained dog was utilized. Due to these limitations, these results are not generalizable to the PD population; however, these researchers feel that the results of this study may reveal the potential negative impact that dog walking can have on the gait pattern of a person with PD.

## 5. Conclusions

Walking with a dog resulted in significant negative changes in gait parameters in people with PD. The results of this study can allow healthcare professionals working with this population to make appropriate recommendations if dogs are part of the patient’s household. From a rehabilitation standpoint, dog owners may have participation-level goals that involve dog walking. A clinician should be aware that the element of dog walking may require additional practice or task-specific training within the rehabilitation setting to ensure safety of this task. The results also bring to light the possible dual-task nature of walking a dog, further supporting the addition of dual-task training within therapy sessions. More studies are needed to assess the effects of dogs who are not service-trained on people with PD. This may improve the safety of the population in order to minimize risk of injury.

## Figures and Tables

**Table 1:** Participant demographic and clinical characteristics.

Demographic Variables	Totals
Gender, No. (%)	Males 15 (79%) Females 4 (21%)
Age, mean (SD *), y ^†^	70.74 (7.19)
Age, range, y	59–78
Hoehn and Yahr Scale	II = 9, III = 10
MDS-UPDRS-III ^‡^, mean (SD *)	27.32 (2.89)
Time since diagnosis, mean (SD *), y ^†^	5.07 (3.87)
Time since diagnosis, range, y ^†^	1–13

* Standard deviation, † Years, ‡ Movement Disorder Society Unified Parkinson’s Disease Rating Scale, motor subsection.

**Table 2:** Changes in gait parameters when walking with a dog (Wilcoxon signed ranks test results).

Gait Parameters	Median (Standard Deviation)		Range				
Variable	ND *	D ^†^	ND *	D ^†^	Effect Size ^‡^	Z	*p*
Left double limb support (%)	27.80 (4.35)	30.00 (3.82)	22.90–40.00	26.50–42.30	0.42	−2.59	0.01 ^§^
Right double limb support (%)	28.20 (4.24)	29.80 (3.84)	23.00–40.60	27.20–42.00	0.47	−2.88	0.004 ^§^
Base of support (cm)	11.53 (3.66)	10.25 (14.46)	5.01–17.49	3.60–70.43	0.35	−2.13	0.03 ^§^
TUG ^‖^ number of steps	13.00 (3.31)	15.33 (3.10)	10.00–24.33	12.67–25.67	0.61	−3.78	0.00 ^§^

* Walking without a dog, ^†^ Walking with a dog, ^‡^ Rank-biserial correlation, ^§^ Statistically significant difference (*p* < 0.05), ^‖^ Timed Up and Go test.

**Table 3:** Changes in gait parameters between walking without and with a dog (paired *t*-test results).

Gait Parameters	Mean (Standard Deviation)		Between-Group Difference		
Variable	ND *	D ^†^	Mean Difference (95% CI ^‡^)	Effect Size ^§^	*p*
Velocity (cm/s)	124.24 (22.56)	113.50 (16.44)	10.74 (4.07 to 17.41)	0.77	0.003 ^‖^
Cadence (steps/min)	112.31 (9.75)	110.23 (11.50)	2.08 (−0.99 to 5.16)	0.33	0.17
Left step length (cm)	66.41 (11.06)	59.31 (11.10)	7.11 (3.83 to 10.39)	1.05	0.000 ^‖^
Right step length (cm)	66.31 (11.01)	63.25 (8.76)	3.05 (.80 to 5.31)	0.65	0.01 ^‖^
Left stride length (cm)	133.13 (20.82)	125.61 (17.34)	7.51 (2.93 to 12.10)	0.79	0.003 ^‖^
Right stride length (cm)	133.60 (20.86)	124.86 (17.80)	8.74 (3.88 to 13.60)	0.87	0.001 ^‖^
Left step time (s)	0.38 (0.03)	0.38 (0.04)	−0.001 (−0.01 to 0.01)	0.00	0.81
Right step time (s)	0.38 (0.04)	0.38 (0.05)	0.002 (−0.01 to 0.002)	0.00	0.77
TUG ^¶^ time (s)	8.44 (2.46)	10.12 (2.48)	−1.67 (−2.07 to −1.28)	2.03	0.00 ^‖^

* Walking without a dog, ^†^ Walking with a dog, ^‡^ Confidence interval, ^§^ Cohen’s *d*, ^‖^ Statistically significant difference (*p* < 0.05), ^¶^ Timed Up and Go test.
